# Metabolic protection by the dietary flavonoid 7,8-dihydroxyflavone requires an intact gut microbiome

**DOI:** 10.3389/fnut.2022.987956

**Published:** 2022-08-17

**Authors:** Priyanka Sharma, Camila Silva, Sarah Pfreundschuh, Hong Ye, Harini Sampath

**Affiliations:** ^1^Department of Nutritional Sciences, Rutgers University, New Brunswick, NJ, United States; ^2^Rutgers Center for Lipid Research, New Jersey Institute for Food, Nutrition, and Health, Rutgers University, New Brunswick, NJ, United States; ^3^Center for Microbiome, Nutrition, and Health, New Jersey Institute for Food, Nutrition, and Health, Rutgers University, New Brunswick, NJ, United States; ^4^Department of Biotechnology, Rutgers University, New Brunswick, NJ, United States

**Keywords:** DHF, microbiome, TrkB, brown adipose tissue, sex effects

## Abstract

**Background:**

7,8-dihydroxyflavone (DHF) is a naturally occurring flavonoid found in *Godmania*, *Tridax*, and *Primula* species that confers protection against high-fat diet (HFD) induced metabolic pathologies selectively in female mice. We have previously reported that this metabolic protection is associated with early and stable remodeling of the intestinal microbiome, evident in female but not male DHF-supplemented mice. Early changes in the gut microbiome in female DHF-fed mice were highly predictive of subsequent metabolic protection, suggesting a causative association between the gut microbiome and the metabolic effects of DHF.

**Objective:**

To investigate a causal association between the gut microbiome and the metabolic effects of DHF using a model of antibiotic-induced gut microbiome ablation.

**Materials and methods:**

Age-matched male and female C57Bl6/J mice were given *ad libitum* access to HFD and drinking water containing vehicle or DHF for 12 weeks. For antibiotic (Abx) treatment, female mice were given drinking water containing a cocktail of antibiotics for 2 weeks prior to HFD feeding and throughout the feeding period. Metabolic phenotyping consisted of longitudinal assessments of body weights, body composition, food, and water intake, as well as measurement of energy expenditure, glucose tolerance, and plasma and hepatic lipids. Protein markers mediating the cellular effects of DHF were assessed in brown adipose tissue (BAT) and skeletal muscle.

**Results:**

Metabolic protection conferred by DHF in female HFD-fed mice was only apparent in the presence of an intact gut microbiome. Abx-treated mice were not protected from HFD-induced obesity by DHF administration. Further, tissue activation of the tropomyosin-related kinase receptor B (TrkB) receptor, which has been attributed to the biological activity of DHF, was lost upon gut microbiome ablation, indicating a requirement for microbial “activation” of DHF for its systemic effects. In addition, we report for the first time that DHF supplementation significantly activates TrkB in BAT of female, but not male, mice uncovering a novel target tissue of DHF. DHF supplementation also increased uncoupling protein 1 (UCP1) and AMP-activated protein kinase (AMPK) protein in BAT, consistent with protection from diet-induced obesity.

**Conclusion:**

These results establish for the first time a requirement for the gut microbiome in mediating the metabolic effects of DHF in female mice and uncover a novel target tissue that may mediate these sexually-dimorphic protective effects.

## Introduction

Dietary flavonoids are non-nutritive bioactive compounds associated with reduced risk for developing numerous pathologies, including obesity, and associated chronic diseases, such as cardiovascular disease, type 2 diabetes, and gastrointestinal disorders (1–4). Flavonoids are poorly absorbed in the upper GI tract, and thus spend a considerable amount of time in the lower GI tract and colon, where their interactions with and biotransformation by gut microbiota (5, 6) are thought to mediate some of their subsequent systemic effects (7).

The composition and diversity of the gut microbiome is influenced by diverse factors such as diet, age, antibiotic use, birth mode, and exercise (8–14). Recent human and animal studies have also highlighted biological sex as a modulator of the gut microbiome and thus as a significant factor influencing disease progression (15–24). For example, sexual dimorphism in the intestinal microbiome appears at the onset of puberty and is thus thought to be driven by the influence of the sex hormones (25, 26). Sexually divergent changes in the gut microbiome have been proposed to predispose males to development of metabolic diseases including diabetes, cardiovascular diseases while conferring protection in pre-menopausal females (27–30). These host–microbiome interactions are thus critical factors modulating metabolic health, but our understanding of how particular dietary components interact with the gut microbiome in the context of biological sex, thereby influencing sexual dimorphism in disease development, is limited.

7,8-Dihydroxyflavone (DHF), is a naturally occurring flavonoid found in several plants, including *Tridax procumbens*, *Godmania aesculifolia*, and *Primula* tree leaves (31–33). DHF is a mimetic of brain-derived neurotrophic factor (BDNF), and like BDNF, it exerts its cellular actions through activation of tropomyosin-related kinase receptor B (TrkB) (32, 34). DHF supplementation has been explored as a therapeutic strategy against neurodegeneration, cognitive decline, and depression (32, 35–37). Recently, we and others have shown that oral DHF supplementation can also protect against high-fat diet (HFD) induced obesity, adiposity, and glucose intolerance (38, 39). Interestingly, this protection was only observed in female but not in male mice, where DHF paradoxically exacerbated lipid accumulation and adipose tissue inflammation. Metabolic protection in female mice was associated with early and stable changes in the composition of the gut microbiome that were highly predictive of subsequent protection against weight gain in female mice.

Given the association between gut microbial changes and body weight, we explored the question of whether the gut microbiome may play a causal role in the metabolic effects attributed to oral DHF. Using a model of antibiotic-mediated gut microbiome ablation, we report here that an intact intestinal microbiome is required to mediate both the cellular signaling and metabolic protection elicited by oral DHF in female mice.

## Materials and methods

### Animal studies

Age-matched male and female C57Bl6/J mice were used in all studies. 13-week old mice were given *ad libitum* access to a HFD (Research Diets D12451; 45% kcals fat, 20% kcals protein, 35% kcals carbohydrate; 5.8% fiber; 4.7 kcal/g metabolizable energy) and drinking water containing vehicle or 1 mg/mL DHF for 12-weeks. Water preparation for the conventional non-antibiotic treated group was as reported previously (38). For antibiotic (Abx) treatment, 11-week old female mice were given drinking water containing a cocktail of Abx including ampicillin (1 g/L), neomycin (1 g/L), metronidazole (1 g/L) (all from Sigma, St. Louis, MO, United States) and vancomycin (VWR Radnor, PA, United States) (0.5 g/L) in 1% sucrose and 0.17% DMSO in PBS for 2 weeks prior to HFD feeding. Bacterial ablation was confirmed as described below. Following this, mice were randomly assigned to Abx-control or Abx-DHF groups and received Abx throughout the 12-week HFD-feeding.

Body weights and food intake were measured weekly, and water intake was measured twice a week (38). Body composition was determined by NMR (Echo MRI, Columbus, OH, United States), at the start and end of HFD-feeding (38). The breeding and care of the animals were in accordance with the protocols approved by the Animal Care and Use Committee of Rutgers University, New Brunswick, New Jersey. For *in vivo* procedures, all efforts were made to minimize discomfort and suffering, in accordance with approved animal care protocols. Following 12 weeks of feeding, mice were euthanized by isoflurane overdose, followed by cardiac exsanguination. Tissues were rapidly collected, snap-frozen in liquid nitrogen, and stored at −80°C until further analyses.

### Gut microbiome ablation

Fresh fecal pellets were collected before and after 2 weeks of Abx-treatment and after 1, 6, and 12 weeks of HFD-feeding and stored at −80°C until further analysis. Fecal bacterial DNA was isolated using QIAamp DNA stool mini kit (QIAGEN, Germantown, MD, United States) following manufacturer’s instructions. Total fecal 16S rRNA was quantified by qPCR using forward primer 785F: 5′-GGA TTA GAT ACC CTG GTA and reverse primer 907R: 5′-CCG TCA ATT CCT TTR AGT TT, spanning the V5 region of 16S rRNA gene (40). A 16S rRNA plasmid DNA with known copy number was used to generate a standard curve to quantify bacterial DNA in samples, as previously described (40).

### Energy expenditure and glucose tolerance test

Energy expenditure was measured by indirect calorimetry (Oxymax, Columbus Instruments, Columbus, OH) after 8 weeks of feeding, as previously described (41). Briefly, oxygen consumption (VO_2_) and carbon dioxide production (VCO_2_) were determined in individually housed animals with *ad libitum* access to HFD and water; both DHF and Abx were maintained in drinking water of respective groups during the calorimetry studies. Animals were acclimatized to Oxymax chambers for 24 h, and VO_2_ and VCO_2_ were recorded for eight consecutive 12-h light and 12-h dark cycles. Metabolic variables were recorded every minute, with a room air reference taken following each cycle of measurements.

Oral glucose tolerance test (GTT) was performed after 10 weeks of HFD-feeding. Briefly, mice were fasted for 4 h, followed by oral gavage of 20% dextrose at a dose of 1 g/kg body weight. Blood was collected from the tail at 0, 20, 40, 90, and 120 min for assessment of plasma glucose using an Accu-Chek glucometer (Accu-Chek Performa, Roche Diagnostics, IN, United States).

### Lipid measurements

Plasma free cholesterol was measured using the Wako Free Cholesterol E Microtiter kit, as per manufacturer’s instructions (FUJIFILM Healthcare, Lexington, MA, United States). Hepatic lipids were extracted by a modified Folch method and separated by thin-layer chromatography (TLC), as previously described (42). Briefly, 20 mg liver tissue was homogenized in chloroform:methanol (2:1), followed by phase separation by the addition of acidified saline and centrifugation. The organic phase was collected, dried under a nitrogen stream, reconstituted in chloroform:methanol (2:1), spotted onto Silica Gel 60 chromatography plates, and developed in heptane:isopropyl ether:acetic acid (60:40:3) until the solvent front reached 1 cm from the top of the plate. Plates were dried, dipped in 10% copper sulfate in 10% phosphoric acid, and charred at 110°C for 30 min.

### Protein immunoblots

Protein samples were prepared by homogenizing tissues in HEPES lysis buffer (50 mM pH 7.4 HEPES, 150 mM NaCl, 10 mM Na-pyrophosphate, 2 mM EDTA, 1% NP-40, 10% Glycerol) containing 1x protease and phosphatase inhibitor cocktail (Sigma-Aldrich). Equal amounts of protein were separated by SDS-PAGE, transferred on PVDF membranes, and immunoblotted with primary antibodies. Primary antibodies including PGC-1α (Novus Biologicals, Littleton, CO, United States), VDAC (Pierce Biotechnology, Waltham, MA, United States), UCP1, COX-IV (Abcam, Cambridge, United Kingdom), and Total TrkB, P-TrkB, PAMPK, Total AMPK, HSP60, SIRT1, and GAPDH (all from Cell Signaling Technology, Danvers, MA, United States) were used at manufacturer recommended dilutions. Following incubation with HRP- or near-IR dye-conjugated secondary antibodies, membranes were visualized using enhanced chemiluminescence or near-IR imaging on an Azure c600 imaging system (Azure Biosystems, United States) and quantified using ImageStudio Lite.

### Statistical analyses

All data are expressed as mean ± SEM. Statistical analysis are carried out using student’s *t*- test for two group comparisons or, for multi-group comparisons using one-way ANOVA, followed by *post hoc* analysis (Bonferroni) in Graph Pad Prism (version 9.3.1 for Windows, GraphPad Software, La Jolla, CA, United States). *p*-values < 0.05 were considered significant.

## Results

### Gut microbiome ablation prevents improvement in metabolism by 7,8-dihydroxyflavone in female mice

We previously reported that mice supplemented with oral DHF for 12 weeks accumulated differential amounts of body weight and fat mass on a high-fat diet (HFD), dependent on sex. Female mice gained significantly less weight and adiposity when supplemented with oral DHF, but male mice were resistant to this metabolic protection afforded by DHF supplementation. We also reported that DHF-induced early and stable remodeling of the gut microbiome in female but not male mice, corresponding with metabolic protection or lack thereof, upon DHF supplementation (38).

To examine a causal role for the gut microbiome in mediating the metabolic effects of DHF in females, we generated an experimental model of gut microbiome ablation by treatment with a cocktail of broad-spectrum antibiotics (Abx) for 2 weeks prior to DHF supplementation and HFD-feeding. Similar protocols have been previously reported to stably and consistently ablate the gut microbial community (43–46). Germ-free mice were not utilized in order to avoid the developmental and immune defects associated with this model (47). Two weeks of Abx-treatment did not result in any body weight changes. Gut microbiome ablation was confirmed by comparing fecal bacterial load at baseline (Week-2) and after 2 weeks of Abx-treatment (Week 0) to a standard curve generated with a 16S rRNA plasmid DNA with known copy number (40). Oral Abx-supplementation decreased the fecal 16S rRNA copy number by >3 log_10_ fold, with similar reductions maintained throughout the course of the study.

**FIGURE 1:**
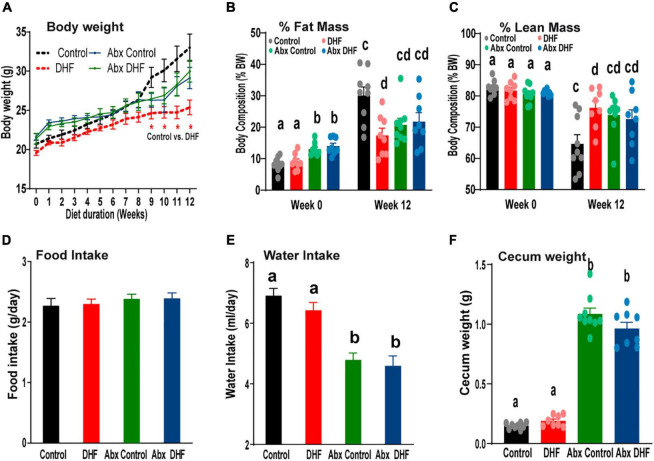
Gut microbiome ablation prevents improvement in metabolism by DHF. **(A–C)** Conventionally raised and Abx-treated females were maintained on a high-fat diet (HFD), supplemented with or without oral 7,8-DHF (1 mg/mL) for 12 weeks. Body weights were measured weekly, and initial and terminal body compositions were measured by NMR. **(D,E)** Food and water intake were measured weekly and biweekly, respectively. **(F)** Cecum weights were measured at the end of 12 weeks. Data were analyzed by one way ANOVA followed by Bonferroni *post hoc* comparison. Groups with different letters are significantly different from each other. *p < 0.05 for Control vs. DHF. Data are expressed as average ± SEM (*n* = 8–9).

Following this, all conventionally raised i.e., which did not receive Abx-treatment and Abx-treated mice received HFD and were segregated into groups receiving vehicle or DHF, with or without oral Abx, for an additional 12 weeks. As we have previously reported (38), DHF treatment reduced weight gain in female mice that were conventionally raised (Figure 1A). Abx-treatment alone has been demonstrated to attenuate weight gain (48–52), independent of changes in food and water intake and solely dependent on absence of gut microbiome. However, Abx-treatment alone did not attenuate HFD-induced weight gain to the same extent as DHF supplementation (Figure 1A). Furthermore, DHF treatment in Abx-treated animals did not have any further impact on body weights (Figure 1A). Similarly, DHF treatment reduced fat mass in conventional mice but had no effect on fat or lean mass in Abx-treated animals (Figures 1B,C).

Food intake was similar across all groups throughout the treatment period (Figure 1D). A significant reduction in water intake was observed with Abx-treatment (Figure 1E), which may be attributable to taste aversion due to antibiotics. This resulted in a daily DHF intake of 0.26 ± 0.01 mg/d/g BW in conventional females and 0.16 ± 0.01 mg/d/g BW in Abx-treated animals. It is important to note that the lower daily dose was still over 2-3-fold higher than a previously reported low-dose of DHF that conferred protection against weight gain in female mice (37, 51). Thus, the lack of protection from weight gain in the Abx-DHF group is not attributable to reduced DHF intake but rather to the concurrent Abx administration. These data indicate that reductions in HFD-induced weight gain due to oral DHF require an intact gut microbiome.

Gut microbiome ablation has been previously reported to significantly increase cecal weights (52–54). These results were recapitulated in the current study, and DHF did not induce any additional effects on cecal weights (Figure 1F).

### Gut microbiome ablation prevents 7,8-dihydroxyflavone-induced attenuation of lipid accumulation

**FIGURE 2:**
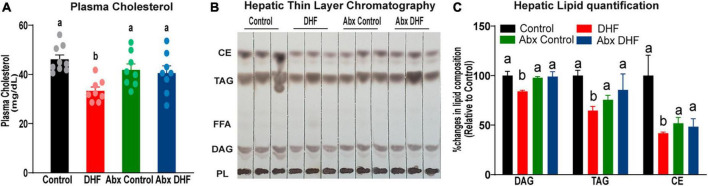
Gut microbiome ablation prevents improvement in lipid metabolism by DHF. **(A)** Plasma free cholesterol was measured at the end of 12 weeks of HFD-feeding in conventional and microbiome-ablated groups. Data are expressed as average ± SEM (*n* = 8–9). **(B)** Hepatic lipids were separated by thin layer chromatography (TLC). CE, cholesterol esters; DAG, diacylglycerols; FFA, free fatty acids; PL, phospholipids; TG, triglycerides. **(C)** Hepatic lipid quantification was done by ImageJ software. Data were analyzed by one-way ANOVA followed by Bonferroni *post hoc* comparison. *p*-values < 0.05 were considered significant. Groups with different letters are significantly different from each other. Data are expressed as average ± SEM (*n* = 3).

Plasma cholesterol was significantly reduced by DHF in the conventional group (Figure 2A). Abx-treatment alone did not reduce plasma cholesterol levels. Importantly, DHF treatment in the Abx-group had no impact on plasma cholesterol, unlike in the conventional group (Figure 2A). Hepatic lipid accumulation was measured by thin-layer chromatography and revealed a significant reduction in levels of diacylglycerols (DAG), triacylglycerols (TAG), and cholesterol esters (CE) in DHF-treated females (Figures 2B,C). However, Abx-DHF animals had no significant reductions in hepatic lipids, relative to Abx-controls. Taken together, these data indicate that reductions in plasma and hepatic lipids by DHF administration require an intact gut microbiome.

### The gut microbiome mediates 7,8-dihydroxyflavone-induced improvements in energy expenditure and glucose tolerance

**FIGURE 3:**
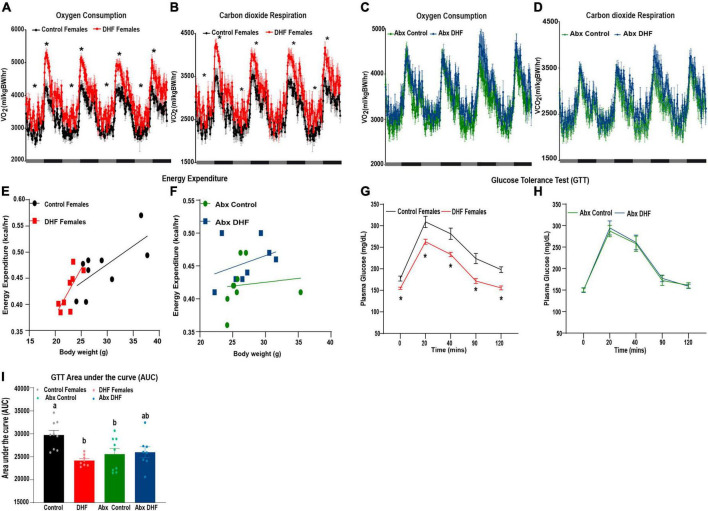
Effect of DHF on energy expenditure and glucose tolerance in conventional and gut microbiome ablated females. **(A,C)** O_2_ consumption, **(B,D)** CO_2_ respiration, and **(E,F)** average cumulative energy expenditure were measured *via* indirect calorimetry over a 4-day period after 8 weeks of HFD-feeding. **(G–I)** Glucose tolerance was measured and area under the curve (AUC) was calculated after 10 weeks of HFD-feeding. Data are expressed as average ± SEM (*n* = 8–9). *p < 0.05 vs. sex-matched controls. Statistical analysis for indirect calorimetry data **(A–F)** was performed by analysis of covariance using web-based analysis tool CalR. Data in panels **(G–I)** were analyzed by one-way ANOVA followed by Bonferroni *post hoc* comparison. Significant differences are identified by * or different letters.

To examine the mechanisms of protection from HFD-induced obesity in DHF-fed females, energy expenditure was measured by indirect calorimetry after 8 weeks of HFD-feeding. Significant increases in O_2_ consumption and CO_2_ respiration were observed in conventionally-raised female DHF-fed mice (Figures 3A,B). Similar increases were not apparent in Abx-treated DHF-fed females (Figures 3C,D). Similarly, regression of energy expenditure against body weight indicated a steeper regression slope in DHF-fed females, relative to control females (Figure 3E; slope: 0.0070 for Control vs. 0.0179 for DHF females) in the conventionally-raised mice, although this difference was not statistically significant. However, this same trend was not observed for DHF-fed females following Abx-treatment (Figure 3F; slope: 0.0011 for Abx-Control vs. 0.0035 for Abx-DHF). We interpret these data to collectively indicate a modest increase in energy expenditure upon DHF supplementation, which is lost in Abx-treated mice. These data are consistent with the protection from weight gain observed in DHF-fed females and lack of protection upon Abx-treatment (Figure 1A) and point to an important role for the gut microbiome in mediating DHF-induced alterations in energy balance.

Consistent with obesity resistance, DHF supplementation of conventional females ameliorated glucose tolerance relative to control females (Figure 3G). Abx-treatment resulted in lower glucose area under the curve (AUC) relative to conventional controls, possibly due to the reduced weight gain in these mice. However, DHF treatment did not improve glucose tolerance in Abx-treated mice, consistent with body weight changes and further implicating a role for the gut microbiome in mediating the improvements in glucose tolerance upon DHF supplementation (Figures 3H,I).

### 7,8-dihydroxyflavone activates tropomyosin-related kinase receptor B in a gut-microbiome dependent manner

7,8-dihydroxyflavone is an activator of tropomyosin-related kinase receptor B (TrkB) which is activated by phosphorylation at discrete tyrosine residues. We measured activation of TrkB by DHF in both male and female mice (32, 34). A previous study reported that oral DHF increased skeletal TrkB phosphorylation in female mice, without any report on the effects of DHF in males (39). Apart from the brain, peripheral expression of TrkB was reported to be greatest in brown and white adipose tissues, with very low expression reported in liver or skeletal muscle (53). Since brown adipose tissue (BAT) plays a critical role in maintaining energy homeostasis, we focused our current investigations on this tissue. Given the prior report on the role of DHF in activating energy expenditure in skeletal muscle, we also investigated the impact of Abx and DHF in gastrocnemius tissue.

**FIGURE 4:**
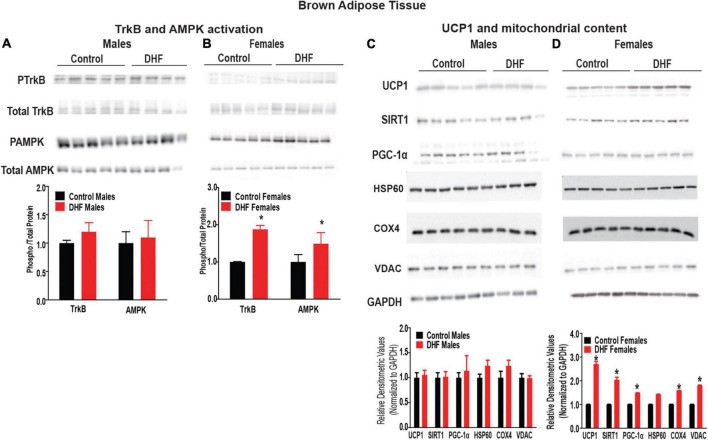
DHF activates TrkB and UCP1 in BAT of females. **(A,B)** Expression and quantification of TrkB and AMPK activation in BAT. **(C,D)** Expression and quantification of mitochondrial content markers in brown adipose tissue (BAT). Statistical analyses were carried out using student’s *t*-test. *p*-values < 0.05 were considered significant. Data are expressed as average ± SEM (*n* = 5).

In BAT, we observed that DHF induced TrkB-phosphorylation in female, but not male mice (Figures 4A,B). Further, phosphorylation and activation of AMP-activated protein kinase (AMPK), one of the downstream targets of TrkB, was also elevated in female but not male mice, consistent with sexually-dimorphic TrkB activation in these tissues (Figures 4A,B). In addition, expression of deacetylase, Sirtuin 1 (SIRT1), and its downstream target Peroxisome proliferator activated receptor-gamma coactivator (PGC-1a), were also increased in BAT of DHF-fed female, but not male mice, indicating increased BAT mitochondrial content and metabolism in these animals (Figure 4C,D). Consistent with activation of SIRT1-PGC-1a signaling, DHF supplementation also significantly increased expression of uncoupling protein-1 (UCP1) in BAT of female but not male mice (Figures 4C,D). Given the role of PGC-1α in modulating mitochondrial mass, we measured expression of mitochondrial content markers including COX-IV, VDAC and HSP60. No differences in mitochondrial content were noted (Figures 4C,D).

**FIGURE 5:**
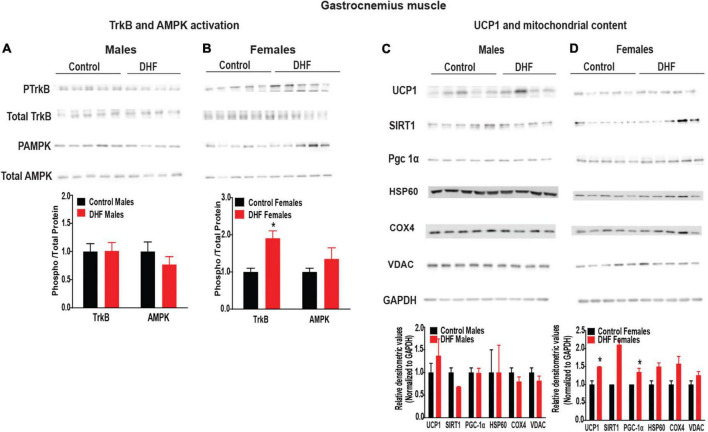
7,8-DHF activates TrkB and UCP1 in gastrocnemius muscle of females. **(A,B)** Expression and quantification of TrkB and AMPK activation in gastrocnemius muscle. **(C,D)** Expression and quantification of mitochondrial content markers in gastrocnemius muscle. Statistical analyses were carried out using student’s *t*-test. *p*-values < 0.05 were considered significant. Data are expressed as average ± SEM (*n* = 5). *p < 0.05 vs. sex-and treatment-matched controls.

Similar to BAT, activation of TrkB was significantly increased in gastrocnemius of female, but not male, DHF-fed mice (Figures 5A,B). While AMPK phosphorylation was significantly elevated in BAT of female DHF-fed mice, only a trend for elevation was observed in female gastrocnemius (Figure 5B). Expression of PGC-1α and SIRT1 were also slightly elevated in gastrocnemius of female mice, but again not to the same extent as observed in BAT (Figure 5D). Since a previous study reported a role for DHF in activating UCP1 in skeletal muscle (39), we measured expression of UCP1 in gastrocnemius, despite its low expression in this tissue. We observed a slight but significant increase in UCP1 expression in gastrocnemius of DHF-fed female mice, but not male mice (Figures 5C,D). Again, this 1.5-fold elevation of UCP1 protein expression in gastrocnemius was not as great as the 3-fold elevation observed in BAT, where the role of UCP1 in modulating energy homeostasis is more clearly established. Similar to BAT, no significant changes in mitochondrial content were evident in gastrocnemius of male or female DHF-fed mice (Figures 5C,D).

Taken together, these data indicate clear increases in TrkB activation, UCP1 induction, and activation of the SIRT1-PGC-1α -AMPK axis in BAT and skeletal muscle of DHF-fed female but not male mice, consistent with the sexually-dimorphic effects of DHF on energy homeostasis and consequently on HFD-induced weight gain.

**FIGURE 6:**
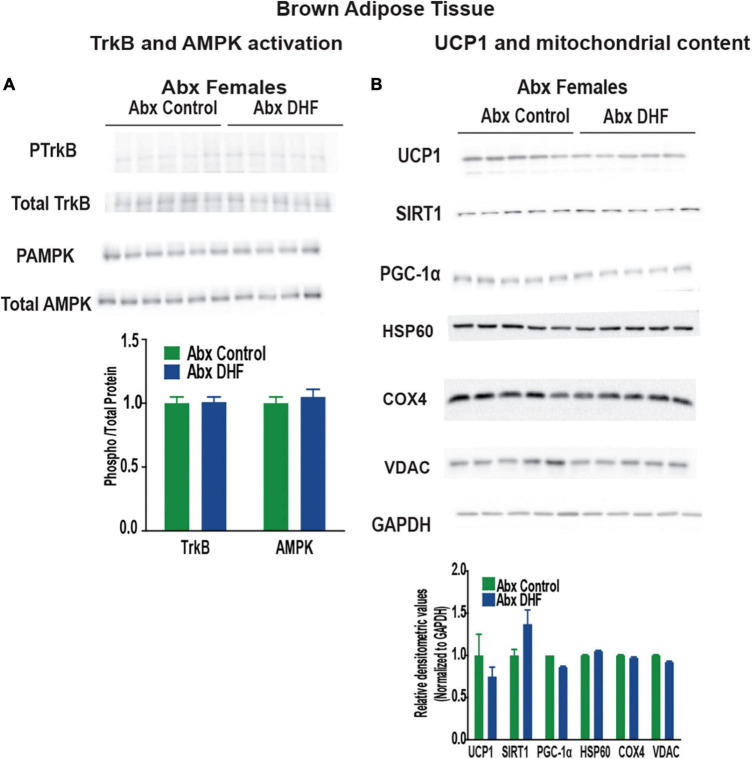
Gut microbiome ablation prevents induction of UCP1 and TrkB activation by DHF in BAT of females. **(A)** Expression and quantification of TrkB and AMPK activation in BAT. **(B)** Expression and quantification of mitochondrial content markers in brown adipose tissue (BAT). Statistical analyses were carried out using student’s *t*-test. *p*-values < 0.05 were considered significant. Data are expressed as average ± SEM (*n* = 5).

**FIGURE 7:**
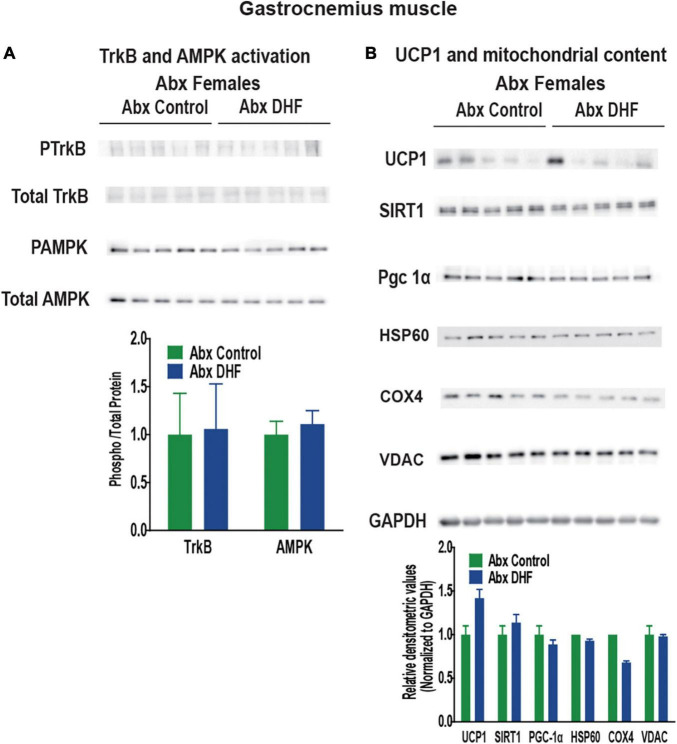
Gut microbiome ablation prevents UCP1 and TrkB activation by DHF in gastrocnemius tissue of females. **(A)** Expression and quantification of TrkB and AMPK activation in gastrocnemius tissue. **(B)** Expression and quantification of mitochondrial content markers in gastrocnemius tissue. Statistical analyses were carried out using student’s *t*-test. *p*-values < 0.05 were considered significant. Data are expressed as average ± SEM (*n* = 5). * *p* < 0.05 vs. sex-and treatment-matched controls.

We next examined these markers in Abx-treated females. Interestingly, upon Abx-treatment, no induction of any of these markers was apparent in either BAT (Figures 6A,B) or gastrocnemius (Figures 7A,B) of female mice. Phosphorylation of TrkB and AMPK, and expression of UCP1, SIRT1, and PGC-1a were static across control and DHF-treated mice upon Abx-treatment. These data clearly indicate, for the first time, that an intact gut microbiome is necessary for induction of TrkB as well as for downstream cellular signaling by DHF.

## Discussion

7,8-dihydroxyflavone is a mimetic of brain-derived neurotrophic factor (BDNF) (31–33, 53–57). Like BDNF, DHF induces dimerization of the tropomyosin-related kinase receptor B (TrkB), followed by activation of downstream signaling molecules, including AKT and ERK (31–33, 39, 53–55, 57–60). However, compared to BDNF, DHF has a much longer half-life of approximately 4-8 h *in vivo* in a primate model (56), compared to just 10 minutes for BDNF (61). This, combined with its shelf-stability and smaller molecular size, make DHF an attractive candidate for clinical applications and oral therapies (31–33, 56, 59, 61–63). Given its actions as a TrkB agonist, many studies regarding DHF have investigated its potential central effects and demonstrated promising results for its use as an antidepressant and neuroprotective factor (34, 36, 58, 64–66) despite known peripheral expression of TrkB receptors in metabolically active tissues (67–69). Furthermore, the prior literature regarding the use of DHF for attenuation of depression and cognitive decline has been carried out almost exclusively in male animals (34, 36, 58, 64–66).

Dietary flavonoids represent a diverse range of polyphenolic compounds that are found in many commonly consumed fruits and vegetables, as well as herbs and grains. Growing evidence suggests that these non-nutritive bioactive components of our diets impart important protective effects against a diverse range of pathologies, including cardiometabolic diseases, specific cancers, and neurocognitive function (5, 70–72). A large proportion of ingested flavonoids are not absorbed in the small intestine but rather reach the colon, where their interaction with colonic bacteria can alter their absorption and excretion rates and give rise to various metabolites that modulate risk for obesity and metabolic disease (73–80). Although some limited reports have suggested sex-differences in flavonoid metabolism, such as *via* glucuronidation, sexually-dimorphic effects of flavonoids on health outcomes have not been systematically investigated (5, 70–72). The lack of such reports may simply stem from the routine reliance on male animals in most studies but serve to seriously limit our understanding of how males and females may respond differentially to therapies intended to modulate the intestinal microbiome. The intestinal microbiome is now understood to be an important determinant of body weight and metabolic health (5, 81–95). In this regard, it is increasingly appreciated that dietary flavonoids, by virtue of their low absorption in the small intestine and greater time spent in the colon, exert important effects on the host intestinal microbiome (5).

We and others have previously reported that oral DHF administration protects female but not male mice from HFD-induced obesity (38, 39). We also reported that paradoxically, DHF supplementation exacerbates some of the metabolic pathologies associated with HFD-feeding in male mice, including increasing hepatic lipid accumulation and adipose tissue inflammation (38). In our previous study, we discovered a novel role for DHF in modulating the gut microbiome of female, but not male, mice at an early time point, prior to any divergence in body weights between DHF-treated and control groups. These stable early changes significantly reduced the relative abundance of pathogenic *Desulfovibrionaceae* ASV_1 and *Rikenella* ASV_41 in females but not males (96–99). *Desulfovibrionceae* are sulfate reducing and lipopolysaccahride (LPS) producing bacteria promoting inflammation and obesity (100, 101). In addition, members of *Rikenella* are also associated with obesity and gut epithelial injury (96, 102). More importantly, these changes in the gut microbiome were highly predictive of subsequent protection from HFD-induced weight gain in female mice, while no such association was observed in male DHF-supplemented animals (38). Given these previous associations between the gut microbiome and the metabolic effects of oral DHF supplementation, the current study was designed to determine whether the gut microbiome is causally linked to the effects of DHF. Using a model of Abx-mediated microbiome ablation, we demonstrate here that an intact gut microbiome is required to mediate the body weight lowering effects of DHF in female mice. While DHF-supplementation reduced body weight and hepatic lipid accumulation in female mice, concurrent Abx-treatment abolished these favorable metabolic effects of DHF. Further, we identified that DHF supplementation induces TrkB activation, AMPK phosphorylation, and induction of SIRT1-PGC-1α and UCP1 in BAT of female mice. Notably, these cellular effects of DHF were absent in male mice, that were not protected from obesity by DHF supplementation. We also report that the effects of DHF supplementation are more apparent in BAT than in skeletal muscle, where more modest effects of DHF supplementation were observed. To our knowledge, these are the first reports of activation of TrkB signaling in BAT by DHF. Given the relatively higher expression of TrkB receptors in BAT than in skeletal muscle (53), we propose that the actions of DHF in this tissue play an important role in mediating the sexually-dimorphic metabolic effects of DHF. Further, we also demonstrate that DHF does not induce any changes in TrkB or associated signaling in BAT or muscle of Abx-treated animals. This lack of cellular changes after Abx-treatment is consistent with the lack of protection from weight gain in this cohort, and thus mechanistically links the gut microbiome to both TrkB signaling in BAT and muscle and the whole-body metabolic effects of DHF.

Peripheral expression of TrkB is highest in BAT in comparison to other metabolically active tissues like liver and skeletal muscle (53). DHF mimics the physiological functions of BDNF by binding and activating its receptor TrkB (53, 59, 60). Although BDNF is known to activate UCP1 and enhance thermogenesis in BAT (103–106), the impact of DHF on BAT was not previously known. Our studies indicate that in addition to TrkB activation, DHF supplementation also impacts AMPK phosphorylation and induction of UCP1, in a gut microbiome-dependent and sexually-dimorphic manner. Other studies have pointed to a potential role for flavonoids in increasing thermogenesis by activating AMPK and downstream mediators including SIRT1 and PGC-1α (107, 108). In our studies, sex- and microbiome-dependent activation of UCP1 expression was associated with improved metabolism in DHF-fed females, suggesting that increased thermogenesis may play a role in preventing obesity and associated metabolic diseases. These effects were observed in skeletal muscle, as well as, to a greater magnitude, in BAT. In this regard, it is important to note that BAT, and skeletal muscle are functionally linked (109) and originate from a common Myf5-expressing precursor cell, distinct from other adipose depots including visceral and subcutaneous fat depots (110). Our observations of sexually-dimorphic activation of TrkB signaling in BAT and gastrocnemius upon DHF supplementation highlight a role for these metabolically-active tissues in mediating the metabolic protection afforded by oral DHF, in a gut microbiome-dependent manner.

The metabolic effects of DHF were recently reported to be impacted by interactions between estrogen receptor-α (ERα) and the TrkB receptor in female mice (111). Specifically, DHF stimulated transactivation of ERα at Serine and Tyrosine residues, while TrkB signaling was reported to mediate ligand-independent activation of ERα. Gut microbiome-mediated alterations in ERα signaling may be an attractive area of future investigations into the mechanism driving the sexually-dimorphic effects of DHF on metabolism. In particular, estrogen has been shown to reduce the incidence of metabolic syndrome *via* alterations of the gut microbiome (112–114). Female and 17β-estradiol-treated male and ovariectomized mice were found to have gut microbiome profiles associated with a lower susceptibility to metabolic endotoxemia and low-grade chronic inflammation, and metabolic syndrome in response to an obesogenic western-diet in comparison to male and ovariectomized control mice. Further, fecal microbiota-transplants from control male mice to female mice transferred the metabolic syndrome phenotype to female mice, while Abx-treatment of males led to loss of the metabolic syndrome phenotype (114). Further, 17β-Estradiol supplementation has been shown to reduce incidence of colorectal cancer, due to alterations in gut microbiome (112). Together, the above findings may warrant exploration of a possible estrogen-mediated link between gut microbial changes elicited by oral DHF and its protective metabolic effects in female mice.

Understanding sexual dimorphism in both gut microbiome composition, as well as response to dietary polyphenols is of great translational relevance. For instance, clinical studies frequently report greater levels of weight loss in male subjects upon dietary and lifestyle interventions relative to female subjects (115–117). Indeed, similar results were observed in placebo-treated subjects in a recent report that examined the combined impact of probiotic therapy with caloric restriction on weight loss. However, upon supplementation with an oral probiotic, weight loss was significantly enhanced in female but not male subjects (118), lending support to the idea that the gut microbiome may differentially impact health in males vs. females. Further studies that examine sexually-dimorphic responses to gut microbiome manipulation will therefore be crucial for the development of personalized nutrition strategies for combating metabolic diseases.

## Data availability statement

The original contributions presented in this study are included in the article/supplementary material, further inquiries can be directed to the corresponding author.

## Ethics statement

The animal study was reviewed and approved by Animal Care and Use Committee of Rutgers University, New Brunswick, NJ, United States.

## Author contributions

PS and HS conceptualized and designed the experiment and conducted data analysis. PS, CS, SP, and HY conducted the animal experiments. PS, SP, and HS prepared the manuscript. HS acquired funding and supervised the study. All authors have read and approved the final version of the manuscript.
